# Abnormal Functional Connectivity Between the Left Medial Superior Frontal Gyrus and Amygdala Underlying Abnormal Emotion and Premature Ejaculation: A Resting State fMRI Study

**DOI:** 10.3389/fnins.2021.704920

**Published:** 2021-07-29

**Authors:** Yan Xu, Xing Zhang, Ziliang Xiang, Qing Wang, Xinfei Huang, Tao Liu, Zhaoxu Yang, Yun Chen, Jianguo Xue, Jianhuai Chen, Jie Yang

**Affiliations:** ^1^Department of Andrology, Jiangsu Province Hospital of Chinese Medicine, Affiliated Hospital of Nanjing University of Chinese Medicine, Nanjing, China; ^2^Department of Andrology, Yangzhou Traditional Chinese Medicine Hospital, Affiliated to Nanjing University of Chinese Medicine, Nanjing, China; ^3^Department of Urology, Jiangsu Provincial People’s Hospital, First Affiliated Hospital of Nanjing Medical University, Nanjing, China; ^4^Department of Urology, People’s Hospital of Xinjiang Kizilsu Kirgiz Autonomous Prefecture, Ürümqi, China

**Keywords:** premature ejaculation, resting-state functional magnetic resonance imaging, functional connectivity, emotion, amygdala

## Abstract

**Introduction:**

Premature ejaculation (PE) is a common sexual dysfunction and is found to be associated with abnormal emotion. The amygdala plays an important role in the processing of emotion. The process of ejaculation is found to be mediated by the frontal-limbic neural circuits. However, the correlations between PE and emotion are still unclear.

**Methods:**

Resting-state functional magnetic resonance imaging (rs-fMRI) data were acquired in 27 PE patients with stable emotion (SPE), 27 PE patients with abnormal emotion (NPE), and 30 healthy controls (HC). We used rs-fMRI to explore the underlying neural mechanisms in SPE, NPE, and HC by measuring the functional connectivity (FC). Differences of FC values among the three groups were compared when choosing bilateral amygdala as the regions of interest (ROIs). We also explored the correlations between the brain regions showing altered FC values and scores of the premature ejaculation diagnostic tool (PEDT)/Eysenck Personality Inventory about neuroticism (EPQ-N) in the PE group.

**Results:**

When the left amygdala was chosen as the ROI, the SPE group exhibited an increased FC between the left medial superior frontal gyrus (SFGmed) and amygdala compared with the NPE or HC group. When the right amygdala was chosen as the ROI, the NPE group exhibited a decreased FC between the left SFGmed and right amygdala compared with the HC group. In addition, FC values of the left SFGmed had positive correlations with PEDT and negative correlations with EPQ-N scores in the PE group. Moreover, FC values of the left superior temporal gyrus had positive correlations with EPQ-N scores in the PE group.

**Conclusion:**

The increased FC values between the left SFGmed and amygdala could reflect a compensatory cortical control mechanism with the effect of stabilized emotion in the limbic regions of PE patients. Abnormal FC between these brain regions could play a critical role in the physiopathology of PE and could help us in dividing PE into more subtypes.

## Introduction

Premature ejaculation (PE) is a common sexual dysfunction with 3% prevalence in Chinese population (Althof S. et al., 2014). According to the International Society for Sexual Medicine (ISSM), a lifelong PE is defined as “ejaculation that always or nearly always occurs before or within about one minute of vaginal penetration” (Althof S.E. et al., 2014; Serefoglu et al., 2014). PE has become a serious problem and arisen important concerns about health because of its influence on the sexual quality of life. It has been found that men with PE constantly suffer from frustration, distress, depression, and inferiority after an unsatisfactory sexual behavior (Waldinger and Schweitzer, 2008; Rowland et al., 2010; Serefoglu et al., 2014). Previous studies showed that the etiology of PE usually included anxiety, penile hypersensitivity, and especially 5-hydroxytryptamine (5-HT) receptor dysfunction (Donatucci, 2006; Rowland et al., 2010; El-Hamd et al., 2019). However, the central pathogenesis of PE is still unclear.

Premature ejaculation was found to be associated with psychological factors, especially abnormal emotion (Zhao et al., 2015; Chen et al., 2017; Jin et al., 2018; Li et al., 2018; El-Hamd et al., 2019). Meanwhile, PE patients were found to suffer from higher levels of partner setbacks, inferiority, or sense of loss (Son et al., 2011; Xia et al., 2016). We suspected that there was a bidirectional relationship and homologous pathophysiologic basis between these two disorders. Resting-state functional magnetic resonance imaging (rs-fMRI) is a useful and non-invasive imaging technique to investigate the central mechanism involved in the regulation of emotion and ejaculatory reflex (Yang X. et al., 2018; Gao et al., 2020a,b). A complex group of brain areas had been found to play an important role in the processing of sexual behavior such as the amygdala, pallidum, thalamus, and caudate nucleus (Kim and Jeong, 2017). According to our previous study, PE patients had a decreased local efficacy and global efficacy in the left amygdala in the white matter brain networks (Chen et al., 2020a). Moreover, negative associations were found between the local efficacy of the left amygdala and the state score of the state-trait anxiety inventory (Morris et al., 2017; Chen et al., 2018). Interestingly, the amygdala was also found to be involved in the emotion perception and processing, which indicated that a dysfunction of the amygdala would lead to abnormal emotion (Goto et al., 2016; Gan et al., 2019; Silverman et al., 2019). In addition, PE patients with depression were found to have an increased nodal betweenness in the right middle frontal gyrus (orbital part) which had been considered to have structural and functional correlations with the amygdala from our previous study (Chen et al., 2020b).

According to previous neuroimaging studies, the volume of the amygdala had been found to have a positive association with sexual drive, and amygdala activity was found to be enhanced when men and women were viewing sexually arousing images (Hamann et al., 2004; Yang L. et al., 2018). The amygdala was highly interconnected with the hypothalamus and electrical stimulation of the amygdala would cause the increasing secretion of gonadotropin in animals (Sundaram et al., 2010; Yang L. et al., 2018). These findings demonstrated that the connection between the amygdala and hypothalamus played an important role in the process of emotion and reproductive neuroendocrine. Moreover, our previous study confirmed that an impaired connectivity of the prefrontal-amygdala pathway could be the underlying pathological basis of the psychogenic erectile dysfunction (Chen et al., 2017). In addition, neuroimaging studies showed that the neural circuit between the orbitofrontal cortex and amygdala was interacted by a series of brain areas instead of being directed by any unitary structure (Morrison et al., 2011; Mehta et al., 2018; Supekar et al., 2018; Zhang et al., 2020). Thus, there existed some cortical or subcortical regions that had interactive effects with the amygdala, which were located in the frontal-limbic circuits.

Emotional stability (neuroticism) is a personality trait that is associated with brain activity during emotion processing, especially in the amygdala (Rampino et al., 2019). Previous studies on anxiety had showed that an inhibitory causal influence existed from the ventromedial prefrontal cortex to the amygdala (Etkin et al., 2009; Mehta et al., 2018). Increased functional connectivity (FC) in the prefrontal cortex-amygdala was found in major depressive disorder patients, which indicated that FC between the amygdala and the prefrontal cortex might provide an underlying indicator of sensitivity to antidepressant medication treatment (Zhang et al., 2020). This result also showed that inhibition of the amygdala could result in negative emotions. Thus, in consideration of the activation of the amygdala in negative emotions, we suspected that the amygdala was not only involved in the process of ejaculatory reflex, but also with the cognitive control over negative emotions and self-referential processing. However, the differences of central mechanisms between emotional disorder and PE were still unclear.

According to a PET-neuro-imaging study, significant changes of regional cerebral blood flow (rCBF) were not being observed in men during ejaculation. Thus, rs-fMRI was chosen to investigate the long-term effects of PE, abnormal emotion on the brain regions, and show their usual working situation (Huynh et al., 2013). In the present study, the subscale of the Eysenck Personality Inventory about neuroticism (EPQ-N) was used to distinguish PE patients into two groups (*N* < 9: SPE, PE with stable emotion; *N* > 14: NPE, PE with abnormal emotion). We hypothesized that SPE and NPE patients could exhibit different FC values in the prefrontal cortex connected with the amygdala. Then, we used the fMRI data to explore the functional connections of the amygdala in these two subtype PE groups using the whole brain FC analysis. Moreover, we explored the associations between FC values of abnormal regions and premature ejaculation diagnostic tool (PEDT) scores, EPQ-N scores in the PE group.

## Materials and Methods

### Participants

A total of 54 right-handed patients with PE were recruited from the Department of Andrology, Jiangsu Province Hospital of Chinese Medicine, Affiliated Hospital of Nanjing University of Chinese Medicine, as well as 30 age and education matched healthy controls (HC; right-handedness). The detailed information about the demographic and clinical features of these participants can be found in Table 1.

**TABLE 1:** Demographic and clinical characteristics of SPE, NPE, and HC.

Characteristics	SPE	NPE	HC	*F*	*P*
Age (year)	29.63 ± 3.81	29.15 ± 6.07	32.07 ± 7.83	1.84	0.17*
Educational level (year)	14.96 ± 2.26	14.67 ± 2.29	14.03 ± 1.63	1.52	0.23*
IIEF-5 (score)	22.33 ± 0.62	22.26 ± 0.66	22.57 ± 0.63	1.85	0.16*
PEDT (score)^*a,b*^	14.41 ± 2.50	15.04 ± 2.61	2.93 ± 2.02	237.82	0.00*
EPQ-N (score)^*c,d*^	6.11 ± 1.89	17.93 ± 2.53	5.73 ± 1.36	343.09	0.00*

*PE, premature ejaculation; SPE, PE with stable emotion; NPE, PE with abnormal emotion; HC, healthy controls; IIEF-5, international index of erectile function; PEDT, premature ejaculation diagnostic tool; EPQ-N, the subscale of Eysenck Personality Inventory about neuroticism.*

*^*a*^Significant differences in the scores of PEDT between SPE and HC.*

*^*b*^Significant differences in the scores of PEDT between NPE and HC.*

*^*c*^Significant differences in the scores of EPQ-N between NPE and HC.*

^*d*^Significant differences in the scores of EPQ-N between NPE and SPE.

*Differences among three groups performed by one-way analysis of variance (ANOVA) test.

In this study, patients were selected based on the following inclusion criteria according to the ISSM PE Guidelines by two experienced andrologists (Althof S.E. et al., 2014): (1) aged from 20 to 45 years; (2) education level >9 years; (3) Intravaginal Ejaculation Latency Time <1 min since the first intercourse; (4) a total score higher than 21 on the international index of erectile function-5 (IIEF-5); (5) a total score higher than 11 on the PEDT; (6) had a stable, heterosexual relationship and regular sexual behavior for at least 6 months; and (7) no current and/or prior use of drugs for PE.

Exclusion criteria for all participants were as follows: (1) any history of serious medical illness and psychiatric or neurological illness; (2) any history of head trauma or loss of consciousness; (3) any history of alcohol dependence or substance abuse; (4) low or loss of sexual desire; (5) with genital abnormalities; and (6) MRI contraindications.

In addition, the test of EPQ was performed to evaluate the stability of emotion. Patients with scores of EPQ-N >14 were classified as the SPE group while patients with scores of EPQ-N <9 were defined as the NPE group.

The present study was approved by the Ethical Committee of Jiangsu Province Hospital of Chinese Medicine, Affiliated Hospital of Nanjing University of Chinese Medicine. Apart from that, written informed consents were obtained from all subjects.

### MRI Data Acquisition

Magnetic resonance imaging (MRI) data were acquired using a 3.0T Siemens Verio scanner at the Affiliated Hospital of Nanjing University of Chinese Medicine. Each participant was positioned comfortably in the scanner and fitted with ear plugs to reduce scanner noise. Prior to scanning, each patient was asked to minimize head and body movements as much as possible while staying awake with eyes closed. The T1-weighted images were obtained in the following scanning parameters: repetition time (TR) = 1,900 ms and echo time (TE) = 2.48 ms, flip angle (FA) = 9°, slice number = 176, slice thickness = 1 mm, field of view (FOV) = 250 mm × 250 mm, voxel size = 1 mm × 1 mm × 1 mm, and scan time = 4 min, 18 s. Resting-state functional images were acquired with the following parameters: TR = 3,000 ms and TE = 40 ms, FA = 90°, slice number = 32, slice thickness/gap = 4/0 mm, FOV = 240 mm × 240 mm, matrix = 64 × 64, 32 axial slices, acquisition time = 6 min, 45 s, resulting in 133 volumes.

### Resting-State Functional Image Preprocessing

**FIGURE 1:**
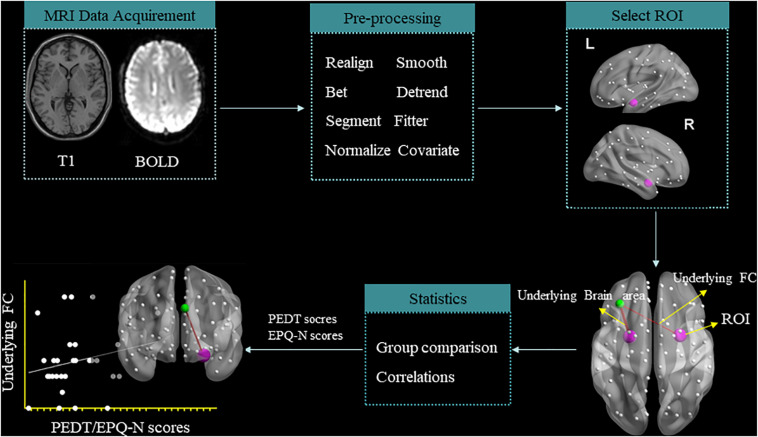
Schematic overview of data acquisition and preprocessing of resting-state functional magnetic resonance imaging.

After image acquisition, preprocessing was performed with the Data Processing Assistant for Brain Imaging (Yan et al., 2016; schematic overview in Figure 1). The first six functional volumes were discarded to allow the participants to get used to the scanner noise and account for the T1 saturation effects. Slice timing and head motion correction and spatial normalization of the remaining 127 volumes were realigned. The remaining data were normalized in the Montreal Neurological Institute (MNI) space, re-sampled with 3 mm × 3 mm × 3 mm resolution, and smoothed with Gaussian kernel (full-width at half maximum = 4 mm). After smoothing, the data underwent temporal filtering (0.01–0.08 Hz) and linear detrending to reduce the influence of the low-frequency drift and high-frequency noise. Furthermore, the nuisance covariates were regressed out from each region, including cerebrospinal fluid signals, global mean signals, white matter signals, and head motion parameters. Individuals with the head motions >2 mm or 2° were discarded from this study. On the basis of our previous studies (Chen et al., 2020a,b), the left amygdala (MNI: *x* = −24, *y* = −1, *z* = −17) and right amygdala (MNI: *x* = 26, *y* = 1, *z* = −18) were selected as regions of interest (ROIs) which were represented by a sphere with a 6-mm radius.

### RS-FC Analysis

*Pearson’s* correlation coefficients between ROIs and the whole brain regions represented the strength of the FC values. The correlation coefficients were converted to *z*-scores with *Fisher’s* r-to-z transform for further analysis. Thus, 54 patients (27 SPE and 27 NPE) and 30 HC were included for FC analysis.

### Statistical Analysis

A one-way analysis of variance (ANOVA) was performed to obtain the FC differences among the three groups by using the Resting-State fMRI Data Analysis Toolkit 1.8 (REST) software. Two sample *t*-test was performed to identify differences between two groups. The threshold was set at *P* < 0.005, and the minimum cluster was 12 voxels. Finally, the *Pearson’s* correlation analysis was used to obtain the correlations between the FC values of the significant brain regions and the PEDT/EPQ-N scores by using the Statistical Package for Social Science (SPSS 23.0) software.

## Results

### Demographic and Clinical Characteristics of the Subjects

As is shown in Table 1, there are no significant differences in age (*F* = 1.8, *p* = 0.17), educational level (*F* = 1.52, *p* = 0.23), and IIEF-5 scores (*F* = 1.85, *p* = 0.16) among the three groups. Both the NPE and SPE groups had higher scores of PEDT than the HC group (*F* = 237.82, *p* = 0.00). NPE group had higher scores of EPQ-N compared with the SPE and HC groups (*F* = 343.09, *p* = 0.00).

### Different FC Values Among the SPE, NPE, and HC Groups

#### Three Groups

##### Left amygdala as ROI

**FIGURE 2:**
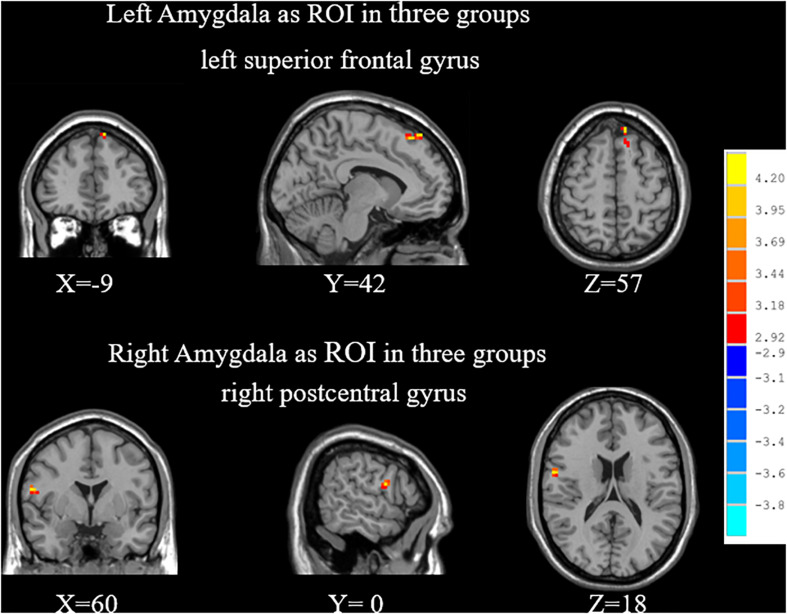
When choosing the amygdala as ROI, significant differences of FC among SPE, NPE, and HC groups. SPE, premature ejaculation with stable emotion; NPE, premature ejaculation with unstable emotion; HC, healthy controls.

By ANOVA analysis, there were significant differences in the FC values between the left amygdala and medial superior frontal gyrus (SFGmed) among groups (Table 2 and Figure 2).

**TABLE 2:** Brain regions showing significant functional connectivity differences among groups.

Brain regions (AAL)		Brodmann area (BA)	Peak MNI coordinates			Cluster	Peak T value
			
			*x*	*y*	*z*		
**Amygdala_L**							
Three groups	Frontal_Sup_Medial_L	8	−9	42	57	19	11.75
SPE vs. NPE	Frontal_Sup_Medial_L	8	−9	36	54	19	3.73
SPE vs. HC	Frontal_Sup_Medial_L	8	−9	42	57	20	4.46
NPE vs. HC	No cluster						
**Amygdala_R**							
Three groups	Postcentral_R	4	60	0	18	15	10.32
SPE vs. NPE	No cluster						
SPE vs. HC	Rolandic_Oper_L	43	−57	−6	9	20	3.79
	Rolandic_Oper_R	4	60	0	18	33	3.85
	Precentral_R	6	42	−6	48	21	3.51
NPE vs. HC	Temporal_Sup_L	22	−63	−24	3	12	3.77
	Postcentral_R	44	60	0	18	13	3.82
	Frontal_Sup_Medial_L	8	0	42	48	20	−3.98

*PE, premature ejaculation; NPE, PE with abnormal emotion; SPE, PE with stable emotion; HC, healthy controls; AAL, anatomic automatic labeling; MNI, Montreal Neurological Institute; *x*, *y*, and *z* were the coordinates of primary peak locations in the MNI space; Amygdala_L, left amygdala; Amygdala_R, right amygdala; Frontal_Sup_Medial_L, left medial superior frontal gyrus; Postcentral_R, right postcentral gyrus; Rolandic_Oper_L, left rolandic operculum; Precentral_R, right precentral gyrus; Temporal_Sup_L, left superior temporal gyrus.*

*The significant level was set at *P* < 0.005 and a cluster size >12 voxels in the ANOVA analysis and *t*-test using the Rest 1.8 program.*

##### Right amygdala as ROI

By ANOVA analysis, there were significant differences in the FC values between the left amygdala and right posterior central gyrus among groups (Table 2 and Figure 2).

#### SPE vs. NPE

##### Left amygdala as ROI

**FIGURE 3:**
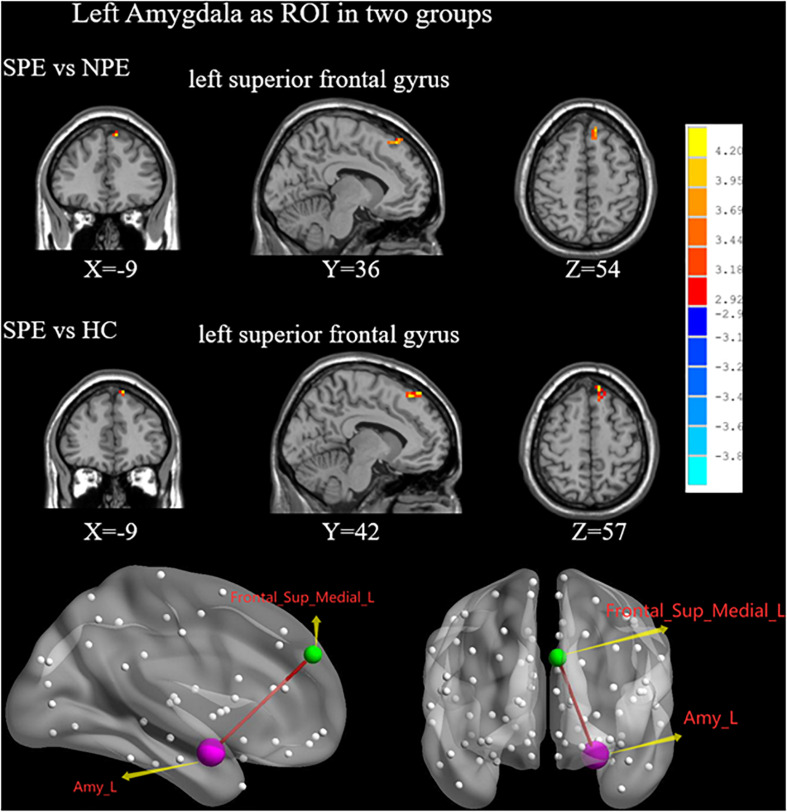
When choosing the left amygdala as ROI, brain regions showing differences of FC values by *t*-test between two groups. SPE, premature ejaculation with stable emotion; NPE, premature ejaculation with unstable emotion; HC, healthy controls. *P* < 0.05 indicated statistically significant differences.

Compared with the NPE group, the SPE group exhibited increased FC values between the left amygdala and SFGmed (Table 2 and Figure 3).

##### Right amygdala as ROI

There were no significant differences in the FC values of the right amygdala between the SPE and NPE groups (Table 2).

#### SPE vs. HC

##### Left amygdala as ROI

Compared with the HC group, the SPE group had increased FC values between the left amygdala and SFGmed (Table 2 and Figure 3).

##### Right amygdala as ROI

**FIGURE 4:**
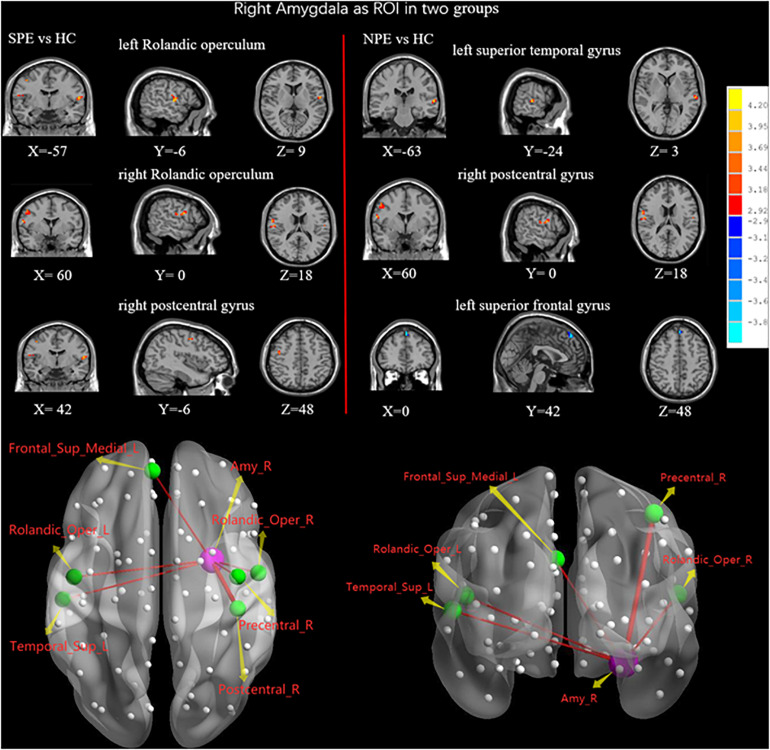
When choosing the right amygdala as ROI, brain regions showing differences of FC values by *t*-test between two groups. SPE, premature ejaculation with stable emotion; NPE, premature ejaculation with unstable emotion; HC, healthy controls. *P* < 0.05 indicated statistically significant differences.

Compared with the HC group, the NPE group had increased FC values between the right amygdala and left Rolandic operculum, right Rolandic operculum, and right precentral gyrus (Table 2 and Figure 4).

#### NPE vs. HC

##### Left amygdala as ROI

There were no significant differences in the FC values of the left amygdala between the NPE and HC groups (Table 2).

##### Right amygdala as ROI

Compared with the HC group, the NPE group had increased FC values between the right amygdala and left superior temporal gyrus (STG), right precentral gyrus. In contrast, the NPE group had decreased FC values between the right amygdala and left SFGmed (Table 2 and Figure 4).

### Correlations Between FC Values and PEDT, EPQ-N Scores in the PE Group

**FIGURE 5:**
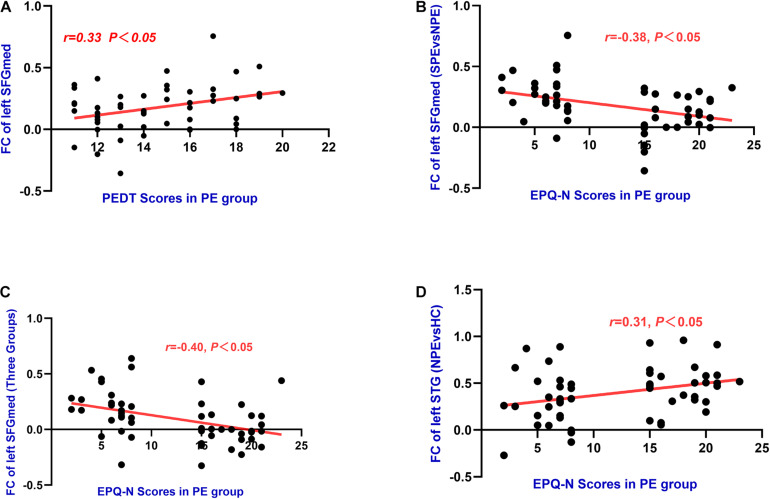
**(A)** The left SFGmed (MNI: *x* = –9, *y* = 36, *z* = 54) had significant positive correlations with PEDT (*r* = 0.33, *P* = 0.02) in PE group. **(B)** The left SFGmed (MNI: *x* = –9, *y* = 36, *z* = 54) had negative correlations with EPQ-N scores (*r* = –0.38, *P* = 0.01) in PE group. **(C)** The left SFGmed (MNI: *x* = –9, *y* = 42, *z* = 57) had negative correlations with EPQ-N scores (*r* = –0.40, *P* = 0.00) in PE group. **(D)** The left STG had positive correlations with EPQ-N scores (*r* = 0.31, *P* = 0.02) in PE group.

Functional connectivity values of the left medial frontal gyrus was found to have positive correlations with PEDT (*r* = 0.33, *P* < 0.05). Meanwhile, FC values of the left SFGmed had negative correlations with the EPQ-N scores in the PE group (MNI: *x* = −9, *y* = 36, *z* = 54: *r* = −0.38, *P* < 0.01; MNI: *x* = −9, *y* = 42, *z* = 57: *r* = −0.40, *P* < 0.01). Moreover, FC values of the left STG had a positive correlation with the EPQ-N scores (*r* = 0.31, *P* < 0.05) in the PE group (Table 3 and Figure 5).

**TABLE 3:** Correlations between FC values and PEDT, EPQ-N scores in PE group.

**Brain regions (AAL)**		**PEDT scores**	**EPQ-N scores**
**Amygdala_L**			
Three groups	Frontal_Sup_Medial_L	*r* = 0.03, *P* = 0.82	*r* = −0.40, *P* = 0.00**
SPE vs. NPE	Frontal_Sup_Medial_L	*r* = 0.33, *P* = 0.02*	*r* = −0.38, *P* = 0.01**
SPE vs. HC	Frontal_Sup_Medial_L	*r* = 0.03, *P* = 0.82	*r* = −0.40, *P* = 0.00**
NPE vs. HC	No cluster		
**Amygdala_R**			
Three groups	Postcentral_R	*r* = 0.22, *P* = 0.11	*r* = 0.11, *P* = 0.44
SPE vs. NPE	No cluster		
SPE vs. HC	Rolandic_Oper_L	*r* = **−**0.20, *P* = 0.15	*r* = −0.23, *P* = 0.10
	Rolandic_Oper_R	*r* = 0.22, *P* = 0.11	*r* = 0.11, *P* = 0.44
	Precentral_R	*r* = 0.02, *P* = 0.89	*r* = −0.08, *P* = 0.44
NPE vs. HC	Temporal_Sup_L	*r* = 0.07, *P* = 0.61	*r* = 0.31, *P* = 0.02*
	Postcentral_R	*r* = 0.02, *P* = 0.89	*r* = 0.11, *P* = 0.44
	Frontal_Sup_Medial_L	*r* = 0.10, *P* = 0.49	*r* = **−**0.22, *P* = 0.17

*PEDT, premature ejaculation diagnostic tool; EPQ-N, the subscale of Eysenck Personality Inventory about neuroticism; PE, premature ejaculation; NPE, PE with abnormal emotion; SPE, PE with stable emotion; HC, healthy controls; Amygdala_L, left amygdala; Amygdala_R, right amygdala; Frontal_Sup_Medial_L, left medial superior frontal gyrus; Postcentral_R, right postcentral gyrus; Rolandic_Oper_L, left rolandic operculum; Temporal_Sup_L, left superior temporal gyrus. Bold values indicated that *P* < 0.05.*

*P* < 0.05 suggested statistically significant differences. **P < 0.01; *P < 0.05.

## Discussion

It is well-known that the amygdala plays an important role in the evaluation of emotional content and the cognitive control of emotion. In this study which bilateral amygdala were regarded as ROIs, the changes of FC values were investigated to explore their relationships with different symptoms of patients. Patients with SPE were found to have increased FC values in the left SFGmed with the left amygdala compared with patients in the HC and NPE groups. Patients with NPE had decreased FC values between the left SFGmed and right amygdala compared with patients in the HC group. Moreover, FC values of the left SFGmed were found to have positive correlations with PEDT scores and negative correlations with EPQ-N scores while the left STG had positive correlations with EPQ-N scores of patients with PE. Therefore, these findings suggested that the increase of FC values could be a compensatory mechanism in response to abnormal emotion caused by PE, the decrease of FC values in the NPE group is probably a decompensation for PE and abnormal emotion.

Sexual behavior was considered as a dialogue of the organism with the external environment and the limbic circuits received or stored the dialogue through the amygdala, then transformed the information in order to output the terminals through the pyramidal system. The medial nucleus of amygdala (MeA) had been proved to play an important role in sexual behavior. Previous studies on rats and gerbils had shown that MeA and bed nucleus of the stria terminalis had enhanced c-Fos expression through olfactory pathways, especially the vomeronasal-accessory olfactory system, which in turn activated the medial preoptic area, and finally led to ejaculation (Veening and Coolen, 1998, 2014; Hull and Dominguez, 2007, 2019; Dhungel et al., 2011).

However, neuroimaging findings were inconsistent. The results of brain activity in human males in a PET-scan study showed that the amygdala was deactivated not only during ejaculation or orgasm, but also during sexual stimulation and erection (Holstege and Huynh, 2011). There was even a similar decrease during romantic love (Bartels and Zeki, 2000). On the contrary, with visual sexual stimulation, activation in the amygdala was found in males and females (Beauregard et al., 2001; Hamann et al., 2004). Moreover, the activation in the amygdala was observed to be suppressed when subjects voluntarily attempted inhibition of sexual arousal (Beauregard et al., 2001; Blair et al., 2007). In studies based on the topographical analysis of diffusion tensor imaging data, there were no significant cerebral macrostructural changes in the amygdala between PE and HC (Atalay et al., 2019), even PE patients had a decreased local efficacy in the left amygdala (Xia et al., 2018). However, previous rs-fMRI studies have found that with erotic visual stimulation, there was more significant activation in the orbital frontal cortex, anterior cingulate gyrus, insula, and amygdala in PE patients (Mouras et al., 2003; Kim et al., 2006; Mallick et al., 2007; Zhang et al., 2017). A study on brain response to visual sexual stimuli in heterosexual and homosexual males showed significant activation in the amygdala, which proved that the amygdala was related to sexual arousal in spite of sexual orientation (Paul et al., 2008). According to the mentioned studies above, the activation of the amygdala could be the reaction of an individual to the stimulus.

In this study, patients with SPE were found to be associated with increased FC values between the left SFGmed and amygdala. In contrast, patients with NPE were found to have decreased FC values between the left SFGmed and right amygdala compared with HC. In a previous study with PET, increased rCBF was found in the left SFGmed while parts of the amygdala were found to be deactivated during ejaculation (Holstege et al., 2003). In another previous study concerning the ejaculatory control center of the brain with a task-based fMRI, an increased neocortical activation was found in the inferior and SFGmed (Beauregard et al., 2001). Meanwhile, a study with MRI to investigate the cerebral macrostructural and microstructural changes between PE patients and HC showed that the bilateral superior frontal cortex had significantly increased thickness and there were no significant cerebral macrostructural changes in the amygdala between PE patients and HC (Atalay et al., 2019). In addition, PE patients were found to have an increased brain current source density of the high beta frequency in both the superior frontal gyri and the right medial frontal cortex after sertraline treatment, which indicated that these regions could constitute parts of the ejaculatory control center of the cerebral neocortex (Kwon et al., 2011). In contrast, another study with EEG showed that the SFGmed in the left hemisphere had a decreased neuronal activation in PE patients under normal conditions, as well as the current source densities of the beta-2 and −3 bands in the right parahippocampal gyrus and the middle temporal gyrus after sexual arousal (Hyun et al., 2008). Moreover, a study based on the anatomical connection pattern and the FC pattern showed that each SFG subregion was involved in two anti-correlated networks including the task positive network and default mode network (DMN). Although the SFGmed had less connection with the DMN, there was a stronger correlation with the part of the brain that was involved in cognitive control (Li et al., 2013).

In addition, previous studies showed that the left hemisphere had a stronger involvement in the regulation of positive emotions while the right hemisphere had a closer relationship with negative emotions (Canli et al., 1998; Compton et al., 2000; Oscar-Berman and Bowirrat, 2005). On the basis of the cerebral lateralization, abnormal FC values in the left hemisphere suggested that the occurrence of PE was more closely related to the impaired processing of positive emotion. This could be due to: (1) the increased activation of the amygdala with sexual stimulation, which enhanced the bottom-up information processing; (2) the SFGmed had a compensatory activation on account of PE, in order to strengthen the top-down control. Our studies confirmed that the left SFGmed was involved in some circuits, which were acted on the inhibition of ejaculation.

According to these previous studies, it was suspected that the increased FC values between the left SFGmed and amygdala were related to PE and emotional stability. These two regions might be involved in the inhibition of ejaculation and the regulation of emotional stability. For SPE, the changes of the left amygdala could lead to the secondary compensatory activation of the left SFGmed, which could help improve the abnormal emotion caused by PE. However, the compensatory activation of the left SFGmed could not improve the symptom of PE. For NPE, the left SFGmed had decreased FC values with the right amygdala. The decreased FC values between the left SFGmed and right amygdala might be a decompensation, which might be associated with the impaired inhibition of ejaculation and regulation of emotional stability.

There were several limitations in this study. Firstly, this was a relatively small sample, cross-sectional study, which might limit the generalization of our results. Secondly, emotion affects hormone level and hormone level affects transduction of synapses. Though we used questionnaires to categorize the emotional level of the participants, physiological data were not acquired. Finally, we have done analysis using *Pearson’s* correlation, while it is known to suffer with limitations (Cole et al., 2016; Aggarwal et al., 2017; Aggarwal and Gupta, 2019). Future studies with a large sample size, detailed physiological data, and better metrics are needed to validate our results.

## Conclusion

The increased FC values between the left SFGmed and amygdala could reflect a compensatory cortical control mechanism with the effect of stabilized emotion in the limbic regions of PE patients. Abnormal FC between these brain regions could play a critical role in the physiopathology of PE and could help us to divide PE into more subtypes.

## Data Availability Statement

The data used to support the findings of this study are available from the corresponding author upon request.

## Ethics Statement

The studies involving human participants were reviewed and approved by the Ethical Committee of Jiangsu Province Hospital of Chinese Medicine, Affiliated Hospital of Nanjing University of Chinese Medicine. The patients/participants provided their written informed consent to participate in this study.

## Author Contributions

JC, JY, and YC designed the experiments. JC, JY, YX, XZ, ZX, QW, XH, TL, ZY, and XJ contributed to clinical data collection and assessment. JC, YX, XZ, and ZX analyzed the results. JC, YX, XZ, and ZX wrote the manuscript. JC and JY approved the final manuscript. All authors contributed to the article and approved the submitted version.

## Conflict of Interest

The authors declare that the research was conducted in the absence of any commercial or financial relationships that could be construed as a potential conflict of interest.

## Publisher’s Note

All claims expressed in this article are solely those of the authors and do not necessarily represent those of their affiliated organizations, or those of the publisher, the editors and the reviewers. Any product that may be evaluated in this article, or claim that may be made by its manufacturer, is not guaranteed or endorsed by the publisher.

## References

[B1] Aggarwal P. Gupta A. (2019). Multivariate graph learning for detecting aberrant connectivity of dynamic brain networks in autism. *Med. Image Anal.* 56 11–25. 10.1016/j.media.2019.05.007 31150935

[B2] Aggarwal P. Gupta A. Garg A. (2017). Multivariate brain network graph identification in functional MRI. *Med. Image Anal.* 42 228–240. 10.1016/j.media.2017.08.007 28866433

[B3] Althof S. McMahon C. Waldinger M. Serefoglu E. Shindel A. Adaikan P. (2014). An update of the International Society of Sexual Medicine’s guidelines for the diagnosis and treatment of premature ejaculation (PE). *J. Sex. Med.* 11 1392–1422. 10.1111/jsm.12504 24848686

[B4] Althof S. E. McMahon C. G. Waldinger M. D. Serefoglu E. C. Shindel A. W. Adaikan P. G. (2014). An update of the international society of sexual medicine’s guidelines for the diagnosis and treatment of Premature Ejaculation (PE). *Sex Med.* 2 60–90. 10.1002/sm2.28 25356302 PMC4184677

[B5] Atalay H. A. Sonkaya A. R. Ozbir S. Culha M. G. Degirmentepe B. Bayraktarli R. (2019). Are there differences in brain morphology in patients with lifelong premature ejaculation? *J. Sex. Med.* 16 992–998. 10.1016/j.jsxm.2019.04.008 31103482

[B6] Bartels A. Zeki S. (2000). The neural basis of romantic love. *Neuroreport* 11 3829–3834. 10.1097/00001756-200011270-00046 11117499

[B7] Beauregard M. Levesque J. Bourgouin P. (2001). Neural correlates of conscious self-regulation of emotion. *J. Neurosci.* 21:RC165. 10.1523/JNEUROSCI.21-18-j0001.2001 PMC6763007 11549754

[B8] Blair K. Smith B. Mitchell D. Morton J. Vythilingam M. Pessoa L. (2007). Modulation of emotion by cognition and cognition by emotion. *Neuroimage* 35 430–440. 10.1016/j.neuroimage.2006.11.048 17239620 PMC1862681

[B9] Canli T. Desmond J. Zhao Z. Glover G. Gabrieli J. (1998). Hemispheric asymmetry for emotional stimuli detected with fMRI. *Neuroreport* 9 3233–3239. 10.1097/00001756-199810050-00019 9831457

[B10] Chen J. Chen Y. Chen G. Dai Y. Yao Z. Lu Q. (2017). Altered brain networks in psychogenic erectile dysfunction: a resting-state fMRI study. *Andrology* 5 1073–1081. 10.1111/andr.12411 29073337

[B11] Chen J. Chen Y. Gao Q. Chen G. Dai Y. Yao Z. (2018). Impaired prefrontal-amygdala pathway, self-reported emotion, and erection in psychogenic erectile dysfunction patients with normal nocturnal erection. *Front. Hum. Neurosci.* 12:157. 10.3389/fnhum.2018.00157 29740301 PMC5928255

[B12] Chen J. Huang X. Lu C. Liu T. Dai Y. Yao Z. (2020a). Graph analysis of DTI-based connectome: decreased local efficiency of subcortical regions in PE patients with high sympathetic activity. *Andrology* 8 400–406. 10.1111/andr.12701 31532583

[B13] Chen J. Yang J. Xiang Z. Huang X. Lu C. Liu S. (2020b). Graph theory analysis reveals premature ejaculation is a brain disorder with altered structural connectivity and depressive symptom: a DTI-based connectome study. *Eur. J. Neurosci*. 53 1905–1921. 10.1111/ejn.15048 33217076

[B14] Cole M. W. Yang G. J. Murray J. D. Repovs G. Anticevic A. (2016). Functional connectivity change as shared signal dynamics. *J. Neurosci. Methods* 259 22–39. 10.1016/j.jneumeth.2015.11.011 26642966 PMC4715953

[B15] Compton R. Heller W. Banich M. Palmieri P. Miller G. (2000). Responding to threat: hemispheric asymmetries and interhemispheric division of input. *Neuropsychology* 14 254–264. 10.1037//0894-4105.14.2.254 10791865

[B16] Dhungel S. Masaoka M. Rai D. Kondo Y. Sakuma Y. (2011). Both olfactory epithelial and vomeronasal inputs are essential for activation of the medial amygdala and preoptic neurons of male rats. *Neuroscience* 199 225–234. 10.1016/j.neuroscience.2011.09.051 21983295

[B17] Donatucci C. (2006). Etiology of ejaculation and pathophysiology of premature ejaculation. *J. Sex. Med.* 3(Suppl. 4), 303–308. 10.1111/j.1743-6109.2006.00305.x 16939474

[B18] El-Hamd M. Saleh R. Majzoub A. (2019). Premature ejaculation: an update on definition and pathophysiology. *Asian J. Androl.* 21 425–432. 10.4103/aja.aja_122_18 30860082 PMC6732885

[B19] Etkin A. Prater K. E. Schatzberg A. F. Menon V. Greicius M. D. (2009). Disrupted amygdalar subregion functional connectivity and evidence of a compensatory network in generalized anxiety disorder. *Arch. Gen. Psychiatry* 66 1361–1372. 10.1001/archgenpsychiatry.2009.104 19996041 PMC12553334

[B20] Gan S. Chen S. Shen X. (2019). The emotion regulation effect of cognitive control is related to depressive state through the mediation of rumination: an ERP study. *PLoS One* 14:e0225285. 10.1371/journal.pone.0225285 31730628 PMC6857946

[B21] Gao M. Feng N. Guo B. Wu J. Sun J. Zhang L. (2020a). Striatum-related intrinsic connectivity deficits in lifelong premature ejaculation patients. *Urology* 143 159–164. 10.1016/j.urology.2020.06.001 32544552

[B22] Gao M. Feng N. Wu J. Sun J. Zhang L. Guo X. (2020b). Altered functional connectivity of hypothalamus in lifelong premature ejaculation patients. *J. Magn. Reson. Imaging* 52 778–784. 10.1002/jmri.27099 32068927

[B23] Goto M. Abe O. Miyati T. Yamasue H. Gomi T. Takeda T. (2016). Head motion and correction methods in resting-state functional MRI. *Magn. Reson. Med. Sci.* 15 178–186. 10.2463/mrms.rev.2015-0060 26701695 PMC5600054

[B24] Hamann S. Herman R. A. Nolan C. L. Wallen K. (2004). Men and women differ in amygdala response to visual sexual stimuli. *Nat. Neurosci.* 7 411–416. 10.1038/nn1208 15004563

[B25] Holstege G. Georgiadis J. R. Paans A. M. Meiners L. C. van der Graaf F. H. Reinders A. A. (2003). Brain activation during human male ejaculation. *J. Neurosci.* 23 9185–9193. 14534252 10.1523/JNEUROSCI.23-27-09185.2003 PMC6740826

[B26] Holstege G. Huynh H. K. (2011). Brain circuits for mating behavior in cats and brain activations and de-activations during sexual stimulation and ejaculation and orgasm in humans. *Horm. Behav.* 59 702–707. 10.1016/j.yhbeh.2011.02.008 21352827

[B27] Hull E. M. Dominguez J. M. (2007). Sexual behavior in male rodents. *Horm. Behav.* 52 45–55. 10.1016/j.yhbeh.2007.03.030 17499249 PMC1952538

[B28] Hull E. M. Dominguez J. M. (2019). Neuroendocrine regulation of male sexual behavior. *Compr. Physiol.* 9 1383–1410. 10.1002/cphy.c180018 31688968

[B29] Huynh H. K. Willemsen A. T. Holstege G. (2013). Female orgasm but not male ejaculation activates the pituitary. A PET-neuro-imaging study. *Neuroimage* 76 178–182. 10.1016/j.neuroimage.2013.03.012 23523775

[B30] Hyun J. S. Kam S. C. Kwon O. Y. (2008). Changes of cerebral current source by audiovisual erotic stimuli in premature ejaculation patients. *J. Sex. Med.* 5 1474–1481. 10.1111/j.1743-6109.2007.00734.x 18194183

[B31] Jin C. Guan M. Dong M. Wu J. He Z. Chen X. (2018). Aberrant baseline brain activity in psychogenic erectile dysfunction patients: a resting state fMRI study. *Brain Imaging Behav.* 12 1393–1404. 10.1007/s11682-017-9805-9 29243122 PMC6290711

[B32] Kim G. W. Jeong G. W. (2017). Menopause-related brain activation patterns during visual sexual arousal in menopausal women: an fMRI pilot study using time-course analysis. *Neuroscience* 343 449–458. 10.1016/j.neuroscience.2016.12.010 27998777

[B33] Kim S. W. Sohn D. W. Cho Y. H. Yang W. S. Lee K. U. Juh R. (2006). Brain activation by visual erotic stimuli in healthy middle aged males. *Int. J. Impot. Res.* 18 452–457. 10.1038/sj.ijir.3901449 16467858

[B34] Kwon O. Y. Kam S. C. Choi J. H. Do J. M. Hyun J. S. (2011). Effects of sertraline on brain current source of the high beta frequency band: analysis of electroencephalography during audiovisual erotic stimulation in males with premature ejaculation. *Int. J. Impot. Res.* 23 213–219. 10.1038/ijir.2011.30 21697858

[B35] Li L. Fan W. Li J. Li Q. Wang J. Fan Y. (2018). Abnormal brain structure as a potential biomarker for venous erectile dysfunction: evidence from multimodal MRI and machine learning. *Eur. Radiol.* 28 3789–3800. 10.1007/s00330-018-5365-7 29600478

[B36] Li W. Qin W. Liu H. Fan L. Wang J. Jiang T. (2013). Subregions of the human superior frontal gyrus and their connections. *Neuroimage* 78 46–58. 10.1016/j.neuroimage.2013.04.011 23587692

[B37] Mallick H. Tandon S. Jagannathan N. Gulia K. Kumar V. (2007). Brain areas activated after ejaculation in healthy young human subjects. *Indian J. Physiol. Pharmacol.* 51 81–85. 17877297

[B38] Mehta N. D. Haroon E. Xu X. Woolwine B. J. Li Z. Felger J. C. (2018). Inflammation negatively correlates with amygdala-ventromedial prefrontal functional connectivity in association with anxiety in patients with depression: preliminary results. *Brain Behav. Immun.* 73 725–730. 10.1016/j.bbi.2018.07.026 30076980 PMC6129411

[B39] Morris L. To B. Baek K. Chang-Webb Y. Mitchell S. Strelchuk D. (2017). Disrupted avoidance learning in functional neurological disorder: implications for harm avoidance theories. *NeuroImage. Clin.* 16 286–294. 10.1016/j.nicl.2017.08.007 28856091 PMC5562176

[B40] Morrison S. E. Saez A. Lau B. Salzman C. D. (2011). Different time courses for learning-related changes in amygdala and orbitofrontal cortex. *Neuron* 71 1127–1140. 10.1016/j.neuron.2011.07.016 21943608 PMC3236094

[B41] Mouras H. Stoléru S. Bittoun J. Glutron D. Pélégrini-Issac M. Paradis A.-L. (2003). Brain processing of visual sexual stimuli in healthy men: a functional magnetic resonance imaging study. *Neuroimage* 20 855–869. 10.1016/s1053-8119(03)00408-7 14568457

[B42] Oscar-Berman M. Bowirrat A. (2005). Genetic influences in emotional dysfunction and alcoholism-related brain damage. *Neuropsychiatr. Dis. Treat.* 1 211–229. 18568071 PMC2416753

[B43] Paul T. Schiffer B. Zwarg T. Krüger T. H. C. Karama S. Schedlowski M. (2008). Brain response to visual sexual stimuli in heterosexual and homosexual males. *Hum. Brain Mapp.* 29 726–735. 10.1002/hbm.20435 17636559 PMC6870890

[B44] Rampino A. Torretta S. Rizzo G. Viscanti G. Quarto T. Gelao B. (2019). Emotional Stability Interacts with Cortisol Levels Before fMRI on Brain Processing of Fearful Faces. *Neuroscience* 416 190–197. 10.1016/j.neuroscience.2019.08.002 31400483

[B45] Rowland D. McMahon C. Abdo C. Chen J. Jannini E. Waldinger M. (2010). Disorders of orgasm and ejaculation in men. *J. Sex. Med.* 7 1668–1686. 10.1111/j.1743-6109.2010.01782.x 20388164

[B46] Serefoglu E. C. McMahon C. G. Waldinger M. D. Althof S. E. Shindel A. Adaikan G. (2014). An evidence-based unified definition of lifelong and acquired premature ejaculation: report of the second international society for sexual medicine ad hoc committee for the definition of premature ejaculation. *Sex. Med.* 2 41–59. 10.1002/sm2.27 25356301 PMC4184676

[B47] Silverman M. Wilson S. Ramsay I. Hunt R. Thomas K. Krueger R. (2019). Trait neuroticism and emotion neurocircuitry: functional magnetic resonance imaging evidence for a failure in emotion regulation. *Dev. Psychopathol.* 31 1085–1099. 10.1017/s0954579419000610 31156078 PMC6620120

[B48] Son H. Song S. Lee J. Paick J. (2011). Relationship between premature ejaculation and depression in Korean males. *J. Sex. Med.* 8 2062–2070. 10.1111/j.1743-6109.2010.02173.x 21235722

[B49] Sundaram T. Jeong G. W. Kim T. H. Kim G. W. Baek H. S. Kang H. K. (2010). Time-course analysis of the neuroanatomical correlates of sexual arousal evoked by erotic video stimuli in healthy males. *Korean J. Radiol.* 11 278–285. 10.3348/kjr.2010.11.3.278 20461181 PMC2864854

[B50] Supekar K. Kochalka J. Schaer M. Wakeman H. Qin S. Padmanabhan A. (2018). Deficits in mesolimbic reward pathway underlie social interaction impairments in children with autism. *Brain* 141 2795–2805. 10.1093/brain/awy191 30016410 PMC6113649

[B51] Veening J. G. Coolen L. M. (1998). Neural activation following sexual behavior in the male and female rat brain. *Behav. Brain Res.* 92 181–193. 10.1016/s0166-4328(97)00190-3 9638960

[B52] Veening J. G. Coolen L. M. (2014). Neural mechanisms of sexual behavior in the male rat: emphasis on ejaculation-related circuits. *Pharmacol. Biochem. Behav.* 121 170–183. 10.1016/j.pbb.2013.12.017 24368305

[B53] Waldinger M. Schweitzer D. (2008). The use of old and recent DSM definitions of premature ejaculation in observational studies: a contribution to the present debate for a new classification of PE in the DSM-V. *J. Sex. Med.* 5 1079–1087. 10.1111/j.1743-6109.2008.00789.x 18331260

[B54] Xia J. Chen J. Yang B. Sun H. Zhu G. Dai Y. (2018). Differences in sympathetic nervous system activity and NMDA receptor levels within the hypothalamic paraventricular nucleus in rats with differential ejaculatory behavior. *Asian J. Androl.* 20 355–359. 10.4103/aja.aja_4_18 29516873 PMC6038171

[B55] Xia Y. Li J. Shan G. Qian H. Wang T. Wu W. (2016). Relationship between premature ejaculation and depression: a PRISMA-compliant systematic review and meta-analysis. *Medicine* 95:e4620. 10.1097/md.0000000000004620 27583879 PMC5008563

[B56] Yan C. Wang X. Zuo X. Zang Y. (2016). DPABI: data processing & analysis for (Resting-State) brain imaging. *Neuroinformatics* 14 339–351. 10.1007/s12021-016-9299-4 27075850

[B57] Yang L. Comninos A. Dhillo W. (2018). Intrinsic links among sex, emotion, and reproduction. *Cell. Mol. Life Sci.* 75 2197–2210. 10.1007/s00018-018-2802-3 29619543 PMC5948280

[B58] Yang X. Gao M. Zhang L. Liu L. Liu P. Sun J. (2018). Central neural correlates during inhibitory control in lifelong premature ejaculation patients. *Front. Hum. Neurosci.* 12:206. 10.3389/fnhum.2018.00206 29872385 PMC5972200

[B59] Zhang A. Yang C. Li G. Wang Y. Liu P. Liu Z. (2020). Functional connectivity of the prefrontal cortex and amygdala is related to depression status in major depressive disorder. *J. Affect. Disord.* 274 897–902. 10.1016/j.jad.2020.05.053 32664030

[B60] Zhang B. Lu J. Xia J. Wang F. Li W. Chen F. (2017). Functional insights into aberrant brain responses and integration in patients with lifelong premature ejaculation. *Sci. Rep.* 7:460. 10.1038/s41598-017-00421-3 28352072 PMC5428429

[B61] Zhao L. Guan M. Zhang X. Karama S. Khundrakpam B. Wang M. (2015). Structural insights into aberrant cortical morphometry and network organization in psychogenic erectile dysfunction. *Hum. Brain Mapp.* 36 4469–4482. 10.1002/hbm.22925 26264575 PMC6869733

